# Supplementary material for: Are carbon emissions trading and green financial instruments synergistic? -Comprehensive quantitative research based on content analysis
Source: PLoS One. 2024 Mar 7;19(3):e0298601. doi: 10.1371/journal.pone.0298601 (PMC10919601; doi:10.1371/journal.pone.0298601)
Supplement: S1 Data — (ZIP) [file pone.0298601.s001.zip › Data/地级市Z_F工作报告.zip!/╜¡╬≈/╛░╡┬╒≥╩╨/└┤╫╘╣1⁄2╓┌║┼í╛┬φ┐╦╩2╛▌═°í┐_2014.docx]

各位代表：

　　现在，我代表市人民政府向大会作工作报告，请予审议，并请各位政协委员和列席会议的同志提出意见。

　　一、2013年工作回顾

　　2013年，是我市经济社会持续发展、项目建设成果丰硕、重点领域改革进展较快的一年，是在新起点上砥砺奋进、激发正能量、谋划新布局的一年。全市上下在省委、省政府和市委的坚强领导下，抢抓机遇，攻坚破难，全面完成市人大批准调整后的主要目标。预计实现生产总值680亿元，增长10.2%；财政总收入92.6亿元，增长10.2%；公共财政预算收入73.8亿元，增长10.7%；固定资产投资538亿元，增长18%；单位生产总值能耗下降3.6%；人口自然增长率低于7.3‰。

　　我们主要做了以下几个方面工作：

　　（一）扩大投入，重大项目取得新突破。全市人民期盼了半个多世纪的浯溪口水利枢纽工程大坝开工建设，标志着项目进入全面施工高峰期。连接京九、皖赣、沪昆三大干线的九景衢铁路正式开工建设，我市连入国家快速铁路网。总投资40亿元的赣东北综合物流园区累计完成投资9亿元，景德镇陶瓷艺术文化交流中心作为2013瓷博会主场馆投入使用，罗家滩收费站西迁主体工程基本完工。总投资25亿元的赣铁城市综合体项目完成前期工作。城市防洪工程等一批重大项目快速推进。跑项争资成效显著，共争取中央资金近9亿元，我市被列入全国地级老工业基地调整改造城市，陶溪川国际陶瓷文化产业园成为2013年全国唯一的非遗保护试点项目。

　　（二）择商选资，开放型经济卓有成效。实际利用外资1.4亿美元，增长19.4%；引进省外5000万元以上项目实际进资248.7亿元，增长20%。坚持“走出去”与“引进来”相结合，积极开展景德镇（香港）重点产业对接恳谈会、直升机产业招商推介会等招商洽谈活动，共签约亿元以上项目50个，总投资233亿元。昌汽公司与世界500强企业北汽集团签订战略重组协议，开启了我市汽车产业新时代。一批落地项目建设加快，投资8亿元的华意压缩新建年产600万台商用压缩机、投资5亿元的陶瓷名坊园等82个5000万元以上项目相继开工；投资4亿元的20兆瓦乐平光伏农业科技大棚、投资5亿元的太阳能电池及配件等53个5000万元以上项目陆续投产。对外贸易取得新进展，生产型外贸企业出口持续增长。

　　（三）做优扶强，工业经济稳中趋好。预计完成规模以上工业增加值220亿元，增长12%。景德镇直升机研发生产基地被科技部认定为国家新型工业化产业示范基地。直升机吕蒙总装园一期扩批项目完成规划和申报，602所无人机研发中心建成使用。江西直升机投资公司市场开拓迈出实质性步伐，生产销售8架整机。预计航空产业主营业务收入120亿元，增长18%。陶瓷产业发展迅速，完成规模以上主营业务收入140亿元，增长22%。产业园区发展加快，高新区、陶瓷工业园和乐平工业园共完成规模以上工业增加值144亿元，增长20%，占全市工业总量65%。重点工业企业不断做强，华意压缩机公司成为全球最大的家用压缩机制造企业。

　　（四）夯实基础，城乡一体化进程加快。预计完成农业总产值78.5亿元，增长4%。粮食总产62万吨，实现“十连增”。完成115座小（二）型病险水库除险加固和6.5万亩高标准农田建设，确保了126.44万亩耕地保有量。新增造林8.6万亩。农业产业化态势良好，市级以上龙头企业达到98家，农民专业合作社增加至745家。镇村联动与和谐秀美乡村建设加快推进，众埠、鹅湖、丽阳3个镇村联动试点镇建设初见成效，景瑶公路沿线综合改造提升工程全面完成，基本完成232个新农村建设点和清洁工程点主要建设项目。

　　（五）发挥优势，服务业发展特色凸显。预计服务业实现增加值227亿元，增长9%，占全市生产总值33.4%。社会消费品零售总额213亿元，增长13%。旅游经济不断做大，接待国内外游客2242万人次，旅游总收入152亿元，分别增长12%、21.3%。古窑成功创建国家5A级景区，紫晶宾馆评为五星级旅游饭店。第十届瓷博会成为历届以来规模最大、人数最多、影响最广的陶瓷盛会。文化产业生机勃发，成功举办“十强文化企业”、“十大文化创新项目”和“十大陶瓷文化品牌”评选活动，申报世界创意城市手工艺之都取得实质性进展。继湖田窑遗址和御窑遗址之后，高岭土遗址也成为国家大遗址。御窑厂入选国家考古遗址公园，新增5 处国家文物保护单位。老城保护区成为省级服务业集聚区，古镇投资公司成为省级服务业龙头企业。金融业发展加快，全市银行业金融机构人民币贷款余额361亿元，比年初增长17.2%。

　　（六）建管并举，城市建设向纵深推进。修编完成了城市总体规划和历史文化名城等专项规划。中心城昌南拓展区与世界500强企业绿地集团达成百亿元战略合作开发协议；引进江西建工集团以BT模式建设道路管网及公建项目，其中投资10亿元的南片区工程正式开工。老城区保护利用工程加快推进，引进国内最大的综合类民营企业、全国500强企业复星集团，投资开发陶溪川国际陶瓷文化产业园；完成一期景观绿化主体工程、天主教堂保护性修缮等项目及16.6万平方米房屋征收；中渡口至瓷都大桥及沿江东路棚户区改造安置房开工建设。依法取缔了群众反映强烈的新村西路露天菜市场。持续打击“两违”，开展了交通拥堵等专项整治。

　　（七）关注民生，群众生活较大改善。预计城镇居民人均可支配收入24000元，增长11%；农民人均纯收入10020元，增长13%。居民消费价格涨幅低于2.6%。新增城镇就业3.9万人，发放小额贷款4.5亿元。保障性住房完成3年累积建设任务，并在工程质量和安全检查评比中名列全省第一。城乡居民社会养老保险参保率95.9%，新农合参合率99.6%，实现养老金网银直发和医保赴昌异地即时结算。开通西安－景德镇－福州航班。与省水投集团战略合作，投资10亿元建设城乡一体化供水项目。实施天然气置换工程，5万户居民用上清洁高效天然气。完成自然村公路115公里路面硬化。2013年市政府为民办10件实事的承诺全部兑现。

　　（八）统筹协调，各项事业全面进步。115个战略性新兴产业和高新技术产业创新项目获国家、省批准立项。景德镇高专成功升本，升格为景德镇学院。市老年大学教学楼新扩建项目基本完工。全市国民人均受教育年限和学前教育入园率均高于全省平均水平。乐平市全国县级公立医院改革试点工作稳步推进。圆满举办十二届市运会暨首届全民健身运动会，成功承办第四届环鄱阳湖国际自行车大赛（景德镇·浮梁站）。“平安瓷都”系列专项行动深入开展，进京访、赴省集体访、重复访数量“三下降”，连续12年未发生重特大安全生产事故。人事编制、统计调查、民族宗教、外事侨务、食品药品、质监、供销、老龄、妇女儿童、残联、人防、档案、地方志等各项工作取得新进步。

　　同时，重点领域改革取得新成效。正式启动实施部分行政区划调整和理顺行政管理体制改革。城市管理体制不断完善，环卫体制改革成功实施，政府服务外包迈出重大步伐。市商业银行组建省级银行取得新进展。建立了统一规范的公共资源交易平台。“营改增”试点和房地产税费一体化征管等财税改革稳步推进。行政审批制度等改革继续深化。这些都为下一步全面深化改革打下了坚实基础。

　　一年来，我们坚持依法行政，自觉接受人大依法监督和政协民主监督，及时办理人大代表意见建议和政协委员提案。严格落实中央“八项规定”，坚持勤政廉政，改进工作作风，提升行政效能，政府自身建设不断加强。

　　各位代表，以上成绩的取得，是全市人民共同努力的结果。在此，我代表市人民政府，感谢全市人民的信任和支持！感谢人大和政协的监督！感谢海内外各界人士对景德镇发展的关心和参与！并向你们表示崇高的敬意！

　　当前，我们在发展中还存在不少困难和问题，主要是：经济总量不大，结构性矛盾依然突出；经济下行压力较大，财政保障能力不强；资金、土地、人才等生产要素短缺，制约发展的瓶颈亟待破解；城市脏乱堵整治任务艰巨，社会治理能力有待提高；政府部分工作人员进取意识、担当意识、创新意识不强，政务环境有待改善。对此，我们将在今后的工作中高度重视，采取措施努力克服和解决。

　　二、2014年工作安排

　　今年政府工作的总体要求是：深入贯彻党的十八大、十八届二中、三中全会及省委十三届七次、八次全会精神，坚持稳中求进的工作总基调，以全面深化改革为统领，打响工业强攻战、招商引资大会战、城市建设攻坚战“三大战役”，着力保障和改善民生，实现经济持续健康较快发展、社会和谐稳定。

　　今年经济社会发展主要预期目标是：生产总值增长10.5%，财政总收入增长13%，公共财政预算收入增长13%，固定资产投资增长20%，规模以上工业增加值增长12.5%，实际利用外资增长10.5%，实际引进内资增长15%，外贸出口6.5亿美元以上，社会消费品零售总额增长13.5%，城镇居民人均可支配收入增长10.5%，农民人均纯收入增长11%，居民消费价格涨幅低于3.5%，单位生产总值能耗下降3.6%，人口自然增长率低于7.29‰。

　　今年重点做好七个方面工作：

　　（一）打响工业强攻战，促进产业转型升级

　　培育壮大主导产业。以昌飞、昌汽、焦化、华意压缩、天新药业、世龙实业、富祥药业、乐华陶瓷等骨干企业为重点，优先发展直升机及通航、汽车及零部件配套制造、陶瓷及文化创意、精细化工及生物医药、制冷压缩机及家电、电子信息等主导产业，努力建设若干个百亿级产业集群和十亿级企业梯队。（1）大力发展航空产业。支持602所、昌飞集团、江西直升机投资公司研发制造，推进昌兴航空装备项目技改和上市，加快引进直升机零部件配套项目，积极争取低空开放试点城市。（2）加快发展汽车及零部件配套制造业。加大力度建设全产业链式新昌河汽车总部基地，推动汽车产业全面转型升级。（3）提升发展陶瓷产业。融观赏性与收藏性为一体，保持艺术陶瓷国际领先地位；强攻高档日用陶瓷和高技术陶瓷；扶持发展高端传统手工制瓷；适度发展卫生建筑用瓷。全力支持汉光陶瓷续建、陶瓷股份公司入园扩建、大瓷庄高档骨质瓷等一批重点项目。（4）升级发展精细化工及医药产业。支持天新药业维生素、金龙化工草甘膦以及宏柏化工扩能增效、开门子药化搬迁扩建等重大项目建设。（5）延长制冷压缩机及家电产业链。全力推进华意压缩新建年产600万台压缩机项目，加快美菱节能冰箱生产线扩建及配套建设。（6）促进电子信息产业创新发展。支持神飞特陶、景光电子等骨干企业技术创新，推动电子信息产业集群发展。

　　做优产业发展平台。科学规划生产力空间布局，围绕206国道沿线，做强高新区、陶瓷工业园、航空科技城、乐平工业园和汽车工业城，全力打造.一线五片.工业走廊。进一步理顺高新区、陶瓷工业园、相关县（市、区）的管理体制及利益关系；优化园区产业布局，加快推进扩园强区；改革园区经营管理体制，探索企业化管理路径；在整合资源的基础上做大园区融资平台，拓宽筹融资渠道；完善园区公共服务和基础设施，推动产城融合发展。（1）充分发挥国家新型工业化直升机产业示范基地作用，支持高新区重点发展直升机总装及航空零部件制造、机械家电、先进装备制造、电子信息等主导产业，力争实现主营业务收入325亿元。（2）充分发挥全国陶瓷产业示范基地作用，加快陶瓷工业园建设，重点推进名坊园续建等项目，着力打造传统日用陶瓷改造提升区、高新技术陶瓷集聚区、手工制瓷基地和传统陶瓷文化集中展示区，力争实现主营业务收入170亿元。（3）充分发挥省级精细化工产业基地作用，加大乐平工业园“三废”治理力度，推进企业技术创新，发展循环经济，力争实现主营业务收入350亿元。

　　坚持绿色生态发展。牢固树立环保优先理念，在加快发展工业的同时，切实保护宝贵的生态环境。加强工业产业类别筛选，科学规划工业发展区域。大力推进企业节能，强化节能考核评价，促进重点工业用能企业能效提升。加强城镇和工业园区污水处理厂及配套管网建设，加快推进清洁生产和资源循环利用，适时开工建设焦化燃气发电二期项目，尽快建成再生资源回收项目，确保工业增加值单位能耗下降5%。

　　（二）推动新一轮大开放，依托大项目促进大发展

　　全面展开招商引资大会战。（1）明确招商方向。主动对接长三角、珠三角，加快融入长江中游城市群，瞄准央企、科企、民企和国内外500强企业，以关联度高、辐射力大、带动力强的龙头型、集群型项目为重点，力争引进2个30亿元以上、55个亿元以上重大产业项目。（2）提升招商质量。更加注重择商选资，引进更多大项目、好项目。打造一流招商团队，组建市直15支专业招商小分队，提高招商专业化水平。创新招商考核手段，严格兑现奖惩。健全招商引资项目准入会商机制，对不符合产业发展规划和过剩产能的项目，坚决拒之门外。（3）优化招商环境。树立“不讲不好办、只讲怎么办”的服务理念，做到“重心前移、包片服务、对口指导”，为投资商提供无微不至、无缝对接的服务，打造生产要素聚集的“洼地”、各类人才向往的“高地”、商务成本较低的.盆地.。同时，进一步扩大对外贸易，大力培育外贸出口企业，鼓励企业开拓国际市场，加快外贸出口转型基地建设，推动加工贸易企业转型升级。

　　千方百计跑项争资。认真研究国家和省有关政策，健全项目动态对接机制，有针对性地申报基础设施和产业项目。积极争取资源型城市第三轮政策延续、国家转型资金等扶持政策，抓好国家服务业试点、低碳试点和生态文明试点城市建设。紧紧抓住省政府打造赣东北开放合作高地机遇，用好政策，包装项目，扩大开放，促进合作。

　　强化基础设施保障能力。落实重大项目市级领导挂点负责和总巡查、总督查、总协调机制。全力推进浯溪口水利枢纽工程，完成河床一次围堰截流。全线开工建设九景衢铁路，启动景德镇北站房和站前广场建设。加快推进城市防洪工程。开工建设昌江吕蒙段防洪堤、飞虹大桥、飞虹大道等园区基础设施项目。完成罗家滩综合管理所及收费站西迁工程。实施国省干线公路改造。建设枫林变电站，完成景东输变电站、天然气置换、垃圾焚烧发电、煤矸石发电等工程。

　　（三）实施城市建设攻坚战，做美中心城区

　　提升城市规划水平。坚持以一流的城市规划引领一流的城市建设，聘请国内外一流专家对城市建成区统一规划设计，突出城市个性，彰显城市魅力。重点完成赣东北综合物流园和陶瓷工业园规划、城市综合交通和大遗址文物保护等专项规划，使城市控制性详规覆盖率达到90%。对珠山中路等主干道两侧建筑外立面及基础设施进行综合改造，形成具有景德镇古镇特色的城市建筑风格。

　　提升城市建设水平。重点推进三大城市板块建设。（1）全力建设中心城昌南拓展区。基本完成中、西片区重点项目征迁和南片区主干道的路面硬化，完成主排水工程、水电气主干管网铺设和综合管沟工程。尽快开工建设市民之家等公建项目和金融中心等商业项目，启动景德镇陶瓷文化旅游城和陶瓷创意中心改造等工程。（2）有序推进老城区保护利用。进一步加大老窑址、老街区、老厂区保护利用力度，老窑址保护重点完成大遗址片区保护规划编制，加快御窑遗址、考古遗址公园和湖田窑遗址文物保护项目建设；老街区保护重点完成“御窑景巷”一期房屋建设和景观绿化，加快实施中渡口至瓷都大桥等棚户区改造项目，推进三闾庙等历史文化街区保护修缮；老厂区保护重点完成陶溪川核心启动区老厂房主体建筑物改造，实施红光、红旗、光明以及刘家弄、落马桥保护利用工程，完成建国瓷厂明清窑作群保护利用工程，推进东城老工厂等棚户区改造项目。（3）加快建设赣东北综合物流园区。推进国大汽车城及物流园二期道路等基础设施建设，启动长途客运中心、公交枢纽中心、旅游集散中心、批发市场等项目建设。同时，完善城市道路设施，建设迎宾大道二期、景东大道二期、方家山保障房规划路及支路、沿江东路及曙光路延伸段等道路，改造拓宽西山路、瓷都大道和吕蒙大桥。

　　提升城市管理水平。（1）在“智慧城市”总体框架内，整合城市防控系统、国土.一张图.等数字化管理资源，构建多元立体数字监管体系。（2）坚持不懈打击“两违”，开展百日整治专项行动，建立根治“两违”长效管理机制。（3）深入开展广告牌匾、渣土运输、占道经营和乱停乱放等综合整治，着力解决城市脏乱堵问题。（4）集约节约用地，严格用地审批，提高土地利用率。（5）启动石岭等“城中村”改造试点。（6）深入开展精神文明主题教育，提高市民法律意识、文明意识、公德意识，引导市民共同呵护美好家园。

　　（四）深化重点领域改革，激发市场主体内生动力

　　根据中央全面深化改革的总体要求和省里的统一部署，结合我市实际，今年重点推进以下改革。（1）抓好政府机构改革，三季度前完成市、县政府机构改革，推进行业协会、商会与行政机关脱钩，加快中介服务机构与行政部门及关联机构的完全脱钩，放开中介服务市场，规范中介服务行为。（2）加快完成部分行政区划调整，同时理顺行政管理体制，形成区划合理、功能完善、管理科学、发展共赢的良好格局。（3）优化企业产权结构，大力发展混合所有制经济，积极推进国有资本、集体资本、非公有资本相互参股控股、合资合作；深化国有企业改革，稳步实施部分重点企业改制重组，继续依法推进停产停业国有企业破产工作；完善国有资产管理体制，全面实行国有企业资产统一监管，加速国有资产流转，优化国有资产资源配置。（4）推进现代服务业改革，围绕开展国家服务业综合改革试点，加强服务业综合协调和管理，完善有利于服务业发展的体制机制。（5）实行警务机制改革，推进指挥调度、应急处突、街面勤务等机制创新，健全情报分析研判、应急指挥处置、警种合成作战等工作机制，提升警务运行效率和服务实战水平。（6）加快科技体制改革，整合科技创新资源，打破条块分割和体制壁垒，构建以企业为主体、市场为导向、政产学研用相结合的技术创新体系。（7）深化城市管理和执法体制改革，进一步完善权责利相统一的市、区两级城市管理机制，向更广领域推行市场化运作模式。

　　同时，深化行政审批制度改革，对上级下放的接收好，对该放手的放彻底，对该管的管到位；深化财税体制改革，建立财权与事权相匹配的财政新机制；推进农村土地流转制度改革，鼓励和支持土地承包向专业大户、家庭农场、农民合作社流转。统筹推进其它各项改革。

　　（五）切实抓好“三农”工作，夯实农业农村发展基础

　　大力发展现代农业。加快农业三大体系建设，实施粮食高产创建项目，确保粮食播种面积139万亩，保障我市粮食安全。提高农业产业化水平，新增6家省级、12家市级农业龙头企业，发展一批农民专业合作社。引导龙头企业向蔬菜、茶叶、养殖等优势特色产业集中，蔬菜播种面积和茶园种植面积分别达到49万亩、14万亩，新建4个标准化规模养殖场。鼓励农业企业参加各类展销活动。

　　加强农村基础设施建设。继续抓好84座小（二）型病险水库除险加固。推进中央小农水重点县和农村河道整治试点县建设，新增和恢复农田灌溉面积8.55万亩。建设高标准农田1.3万亩。实施“森林城乡、绿色通道”工程，全年造林4万亩。防控森林火灾和林业有害生物。加强防汛安全隐患排查和山洪地质灾害防治，确保安全度汛。

　　建设和谐秀美乡村。把镇村联动作为城乡发展一体化的关键环节来抓，重点建设7个中心镇、特色镇及100个中心村、特色村、“城中村”，抓好3条美丽乡村综合示范带联动建设。继续向省申报并抓好200个左右省级新农村建设点和农村清洁工程点建设。做好浯溪口库区移民搬迁工作，加快建设峙滩新集镇。推进新农村现代流通供销合作网络工程，力争覆盖率达到60%。积极实施改善农村人居环境行动计划。

　　发展壮大县域经济。实施工业转型升级、现代农业“接二连三”、服务业倍增、县城建设提升、镇村联动发展、城乡环境治理“六大工程”，积极探索“城乡统筹、产城融合、区域一体、各具特色”的县域经济发展路子。支持和引导各县（市、区）根据资源禀赋和产业基础，进一步完善产业布局和发展规划，实现特色发展和差异化发展。

　　（六）壮大提升现代服务业，增强经济发展活力

　　着力打造旅游强市。围绕建设国际旅游名城的目标，促进旅游资源由分散开发向整体开放开发推进、旅游消费由观光旅游为主向休闲度假观光并重、旅游收入由门票为主向综合收入转型升级，力争实现旅游总收入195亿元， 增长28.4% 。（1）突出旅游发展重点。编制景德镇国际旅游名城发展规划，优先发展城市旅游和陶瓷文化旅游，打造老城核心旅游产业区，兼顾发展乡村旅游、山水生态游和红色旅游等。（2）加大创建力度。支持高岭·瑶里创建国家5A级景区、国家生态旅游示范区和国家旅游度假区，启动陶瓷工业园、陶溪川国际陶瓷文化产业园创建旅游产业园区。（3）加强旅游宣传和营销，培育旅游地接市场。（4）创新旅游发展机制。加快理顺市县两级旅游行政管理体制，构建政府主导型、企业专业化经营的大旅游发展格局。筹建景德镇旅游集团，支持浮梁县开展旅游综合改革试点。

　　大力发展金融服务业。进一步完善金融机构体系，积极引进金融机构，大力推进地方金融机构发展，加快市商业银行组建省级法人银行，鼓励社会资本参与组建中小型银行、村镇银行、小额贷款公司等新型金融机构。充分发挥银行信贷市场融资主渠道作用，大力推进直接融资，鼓励企业通过发行企业债、私募债等融资工具募集资金，支持有条件的企业上市融资。加快组建景德镇陶瓷交易所。鼓励县（市、区）、重点企业以及民间资本组建融资担保公司。

　　积极发展其他现代服务业。以培育和引进服务业龙头企业为重点，繁荣文化创意产业，推动服务业集聚区建设。加快培育电子商务平台，大力支持电子商务发展。提升瓷博会办展效益和水平，打造会展经济品牌。积极发展商务楼宇经济。大力发展现代物流、科技服务、信息服务等生产性服务业，加快发展文化娱乐、居家养老、医疗保健、家政服务等生活性服务业。

　　（七）着力保障和改善民生，统筹社会事业协调发展

　　加大民生保障力度。各级财政安排民生资金76.8亿元，实施民生工程，重点为民办好10件实事： （1）为城乡困难群众购买公益性就业岗位900个；新增小额贷款3.5亿元。（2）城市和农村低保标准分别提高到450元、230元，人均月补差金额分别增加到270元、145元。（3）提高城乡独生子女伤残、失独家庭特别扶助金标准，每人每月增加60元。（4）开工建设保障性住房2350套，改造各类棚户区3516户，发放廉租补贴9100户。（5）完成城区1.8万户自来水一户一表改造，解决3万农村人口饮水安全问题。（6）建设2个地下人行通道，规划建设4个停车场，安装8条道路路灯，对老城区10条道路和排水系统进行改造。（7）建设、改造8个街心花园（公园）。（8）改造3个农贸市场。（9）建设15座站厕合一型垃圾中转站。（10）新增30辆以上空调公交车和100辆出租车。

　　协调发展各项社会事业。更加注重科技协同创新，提升企业创新发展能力。创建学前教育省级示范园，加快省级职业教育示范校和农村义务教育学校标准化建设，优化城区义务教育资源布局。继续推进公立医院改革，抓好公共卫生服务项目，完善城乡社区公共卫生和城市医疗服务体系，强化食品药品监管。完善公共文化基础设施，完成陶瓷博物馆改建。推进景德镇申报世界文化遗产，力争申报世界创意城市手工艺之都取得成功，办好“中国文化遗产日”主场城市活动。推进省级体育强市建设，积极迎战第十四届省运会。

　　提高社会治理水平。深入推进“平安瓷都”建设，完善立体化社会治安防控体系，夯实治安防控基层基础，落实属地涉事单位责任，及时化解矛盾纠纷，维护群众合法权益。完善领导包案制、信访三级终结制、来访接待中心联合接访制，推进网上信访办理系统和视频接访系统全覆盖。加强网络监管和舆论引导，净化网络环境。建立健全事故防控及安全生产责任体系，完善应急处理机制，防范和遏制重特大安全事故。

　　各位代表，今年是贯彻落实党的十八届三中全会精神、全面深化改革的开局之年，是实施“三大战役”的第一年。做好今年的工作，事关全面深化改革能否良好开局，事关“三大战役”能否首战告捷，事关市委、市政府确定的战略目标能否最终实现，意义十分重大，也更加凸显加强政府自身建设的重要性与紧迫性。为此，我们必须进一步解放思想。不断突破封闭僵化的思想桎梏，最大限度点燃创新创造的激情，以思想大解放推动生产力和社会活力的大解放，在全面深化改革中提振精神，在激发活力中比拼才干。必须坚持依法行政。深入推进网上审批和电子监察系统建设，开展并联审批和项目审批代办服务工作，扩大政务公开，优化政务环境，努力做到不“越位”、不“缺位”、不“错位”。必须切实提高执行力。将执行力作为政府工作的生命力来抓，进一步增强大局意识、担当意识、进取意识和竞争意识，做到言必信、行必果，不断提高执行力、操作力、创新力和破解难题的能力。必须坚持廉洁从政。以开展群众路线教育实践活动为契机，进一步转变作风，落实“八项规定”和反对“四风”的各项要求，建设清廉政府。

　　各位代表，我们已迈入充满希望的新一年，开始了充满挑战的新征程。让我们在省委、省政府和市委的坚强领导下，紧紧依靠全市人民，下定决心抓改革，凝心聚力促发展，全心全意惠民生，为实现景德镇科学发展新跨越、谱写全面建成小康社会新篇章而努力奋斗！
